# Supplementary figures and images for: Soma-axon coupling configurations that enhance neuronal coincidence detection
Source: PLoS Comput Biol. 2019 Mar 4;15(3):e1006476. doi: 10.1371/journal.pcbi.1006476 (PMC6417746; doi:10.1371/journal.pcbi.1006476)

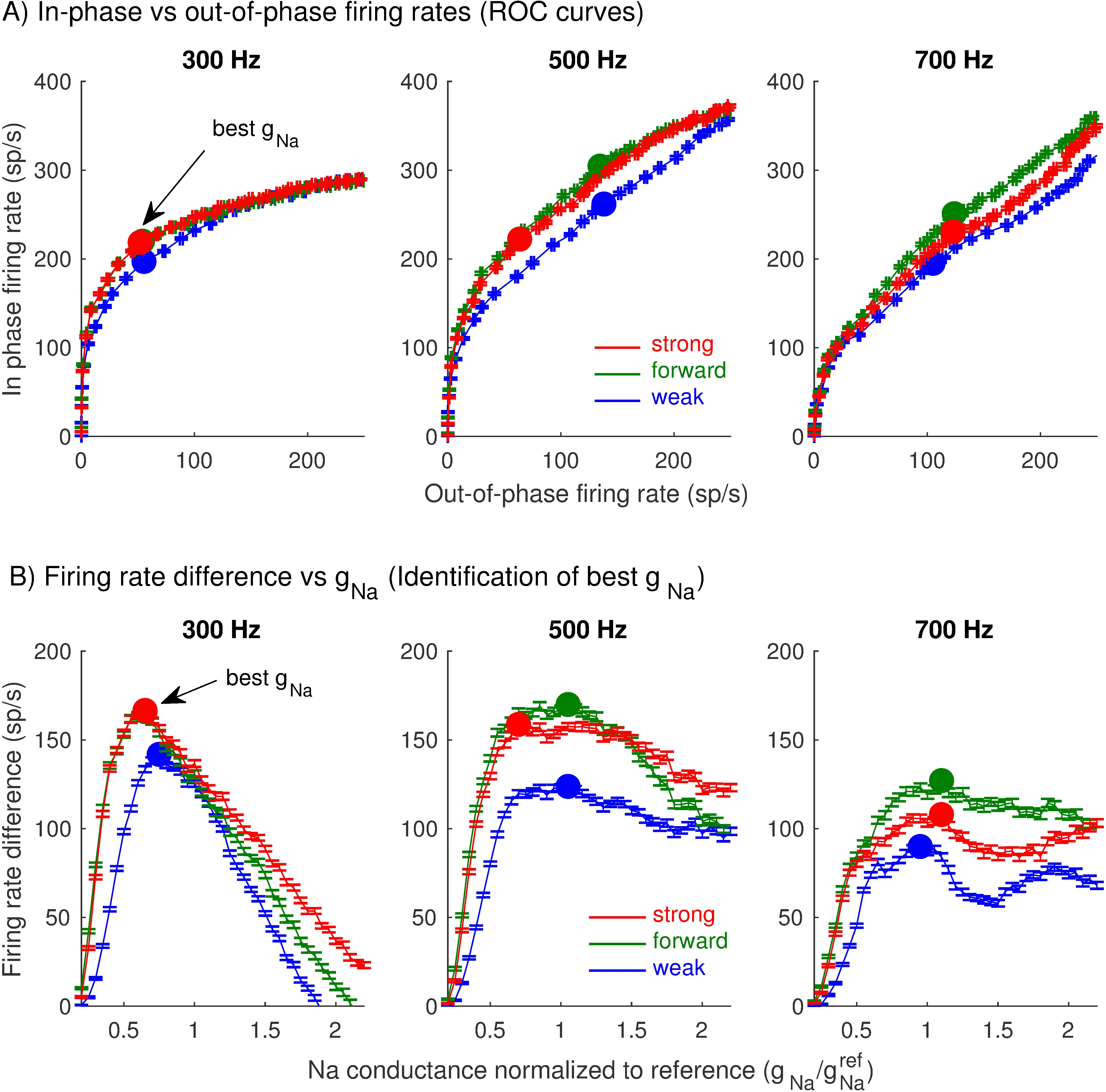

Supplement: S1 Fig — A: Firing rate to in-phase inputs (y-axis) plotted against firing rate to out-of-phase inputs (x-axis) with gNa varied to explore a range of firing rates. B: Firing rate differences (y-axis) plotted against Na conductance (gNa normalized to reference gNa values, see text for details). We identify the value of gNa that is best for coincidence detection by finding the gNa value that results in the greatest firing rate difference. Best gNa and the corresponding firing rate values are marked with dots in these figures. Error bars are standard error of mean firing rates computed from 100 repetitions of 1-second long simulations for each gNa and frequency. We show results for the weakly-coupled, forward-coupled, and strongly-coupled models (colored lines) and responses to three stimulus frequencies (from left to right: 300, 500, and 700 Hz). (TIF) [file pcbi.1006476.s001.tif]

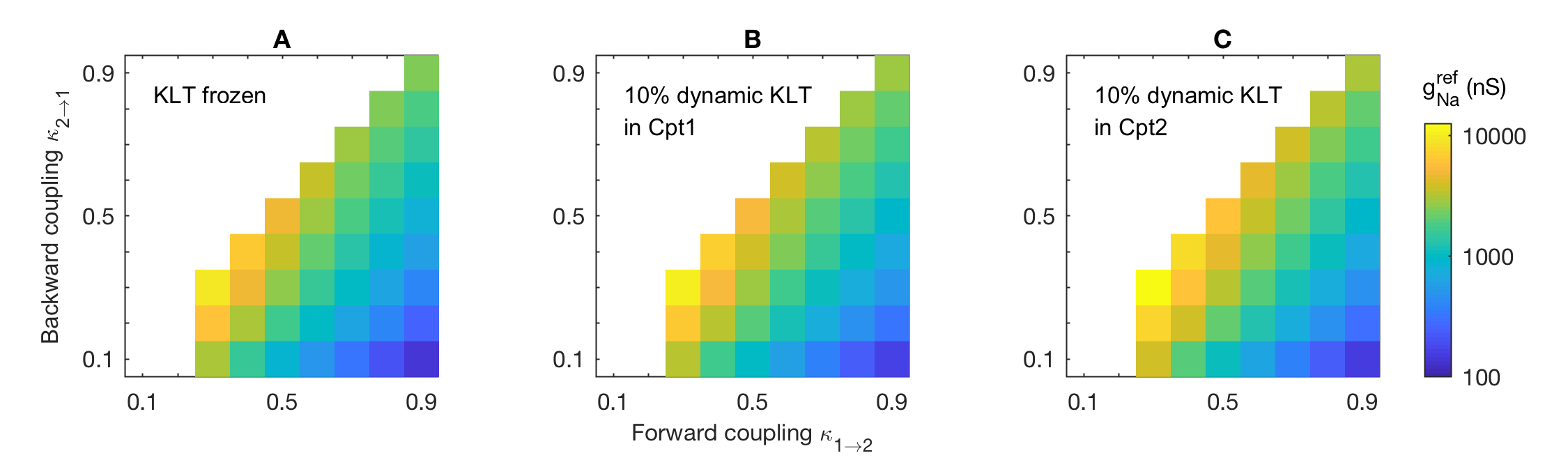

Supplement: S2 Fig — A: Reference gNa across parameter space of coupling strengths, for model without dynamic KLT. This panel reproduces Fig 2B, see text for definition of reference gNa. B: Reference gNa for two-compartment models with 10% of leak conductance in Cpt1 replaced by dynamic KLT conductance. C: Reference gNa for two-compartment models with 10% of leak conductance in Cpt2 replaced by dynamic KLT conductance. (TIF) [file pcbi.1006476.s002.tif]

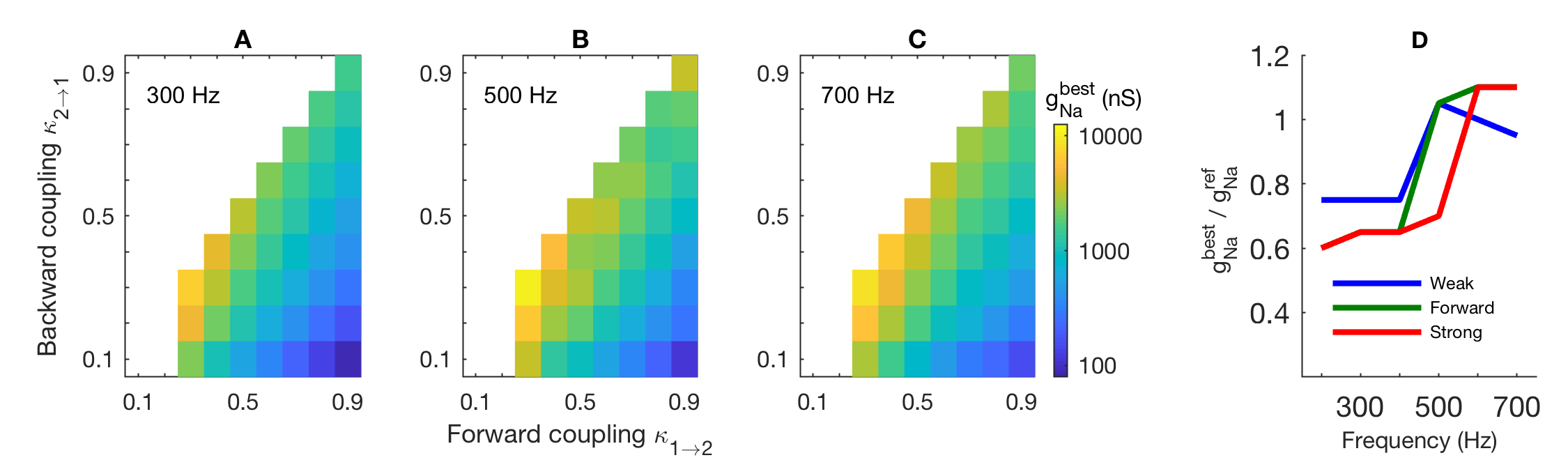

Supplement: S3 Fig — The value of gNa at which the two-compartment model achieves its best coincidence detection sensitivity (maximal firing rate difference between response to in-phase and out-of-phase inputs) for (A) 300 Hz stimuli, (B) 500 Hz stimuli, and (C) 700 Hz stimuli. D: Detailed view of these best gNa values for the weakly-coupled, forward-coupled, and strongly-coupled models as function of input frequency. (TIF) [file pcbi.1006476.s003.tif]

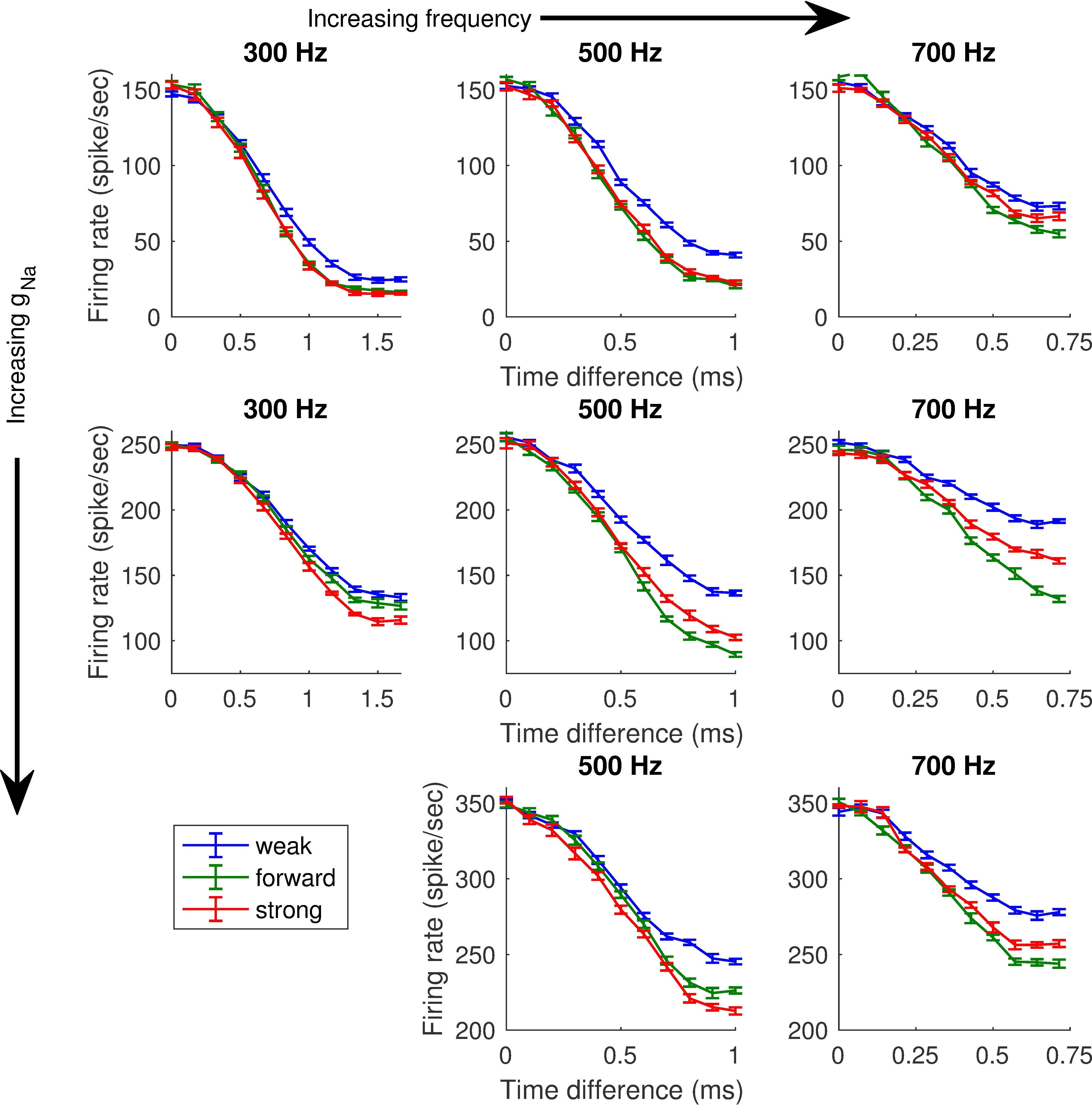

Supplement: S4 Fig — Firing rates in response to 300 Hz (left column), 500 Hz (middle column) and 700 Hz (right column) stimuli. Sodium conductance values are selected so that the firing rate for coincident inputs (0 ms time difference) for each model is 150 spikes/second (top row), 250 spikes/second (middle row), or 350 spikes/second (bottom row). (TIF) [file pcbi.1006476.s004.tif]
